# A Simple Manual Maneuver for Suspected Back‐Patch‐Related Positional Alerts During Pulsed Field Ablation

**DOI:** 10.1002/joa3.70470

**Published:** 2026-09-21

**Authors:** Saki Yamano, Tetsuma Kawaji, Misaki Naka, Ryuta Ishida, Masaya Kitamori, Masashi Kato, Takafumi Yokomatsu, Shinji Miki

**Affiliations:** ^1^ Department of Cardiology Mitsubishi Kyoto Hospital Kyoto Japan; ^2^ Department of Cardiovascular Medicine, Graduate School of Medicine Kyoto University Kyoto Japan

**Keywords:** atrial fibrillation, back patch displacement, CARTO system, positional alert, pulsed field ablation

## Abstract

Summary of the manual counter‐traction maneuver for suspected back‐patch‐related positional alerts during PFA. Cough‐related body movement caused suspected cranial‐side back‐patch displacement and triggered CARTO positional alerts. Light cranial skin counter‐traction was applied opposite to the apparent patch‐shift direction without direct contact with the patch. Among 23 alert episodes, 22 were resolved, with a median correction time of 1.62 s and a median measured displacement of 11.69 mm. Pre‐ and post‐alert anatomical maps showed no gross mismatch on visual comparison, although small localization errors cannot be excluded.

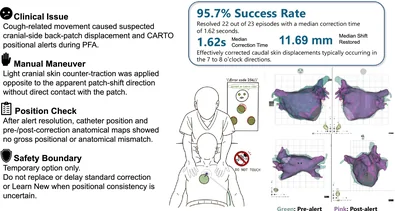

An 82‐year‐old woman with paroxysmal atrial fibrillation underwent pulmonary vein isolation using the PulseSelect pulsed field ablation (PFA) system (Medtronic, Minneapolis, MN, USA), guided by the CARTO 3 electroanatomical mapping system (Johnson & Johnson, New Brunswick, NJ, USA) [1, 2]. The procedure was performed under deep sedation with intravenous propofol, and airway management was achieved using a supraglottic airway device. The shoulders and chest were secured with fixation straps.

During the approach to the right inferior pulmonary vein, a strong cough caused sudden body movement. Because the torso was firmly secured by the fixation straps, this abrupt movement forced the skin on the patient's back to slide caudally relative to the procedure table, resulting in the displacement of the posterior back patch and triggering a positional alert (Error Code 256) (Figure 1A). This alert was related to the body reference system. If standard correction is unsuccessful, reacquisition of the body reference using the Learn New function may be required, which interrupts workflow and can invalidate prior positional information. While this phenomenon represents a general mapping‐system issue related to reference patch displacement rather than a system‐specific error of PFA, it remains highly clinically relevant. PFA energy delivery can inherently stimulate adjacent nerve or muscle tissue, frequently provoking coughing or diaphragmatic contractions [3]. Thus, CARTO‐guided PFA procedures are particularly susceptible to such reference shifts.

**FIGURE 1:**
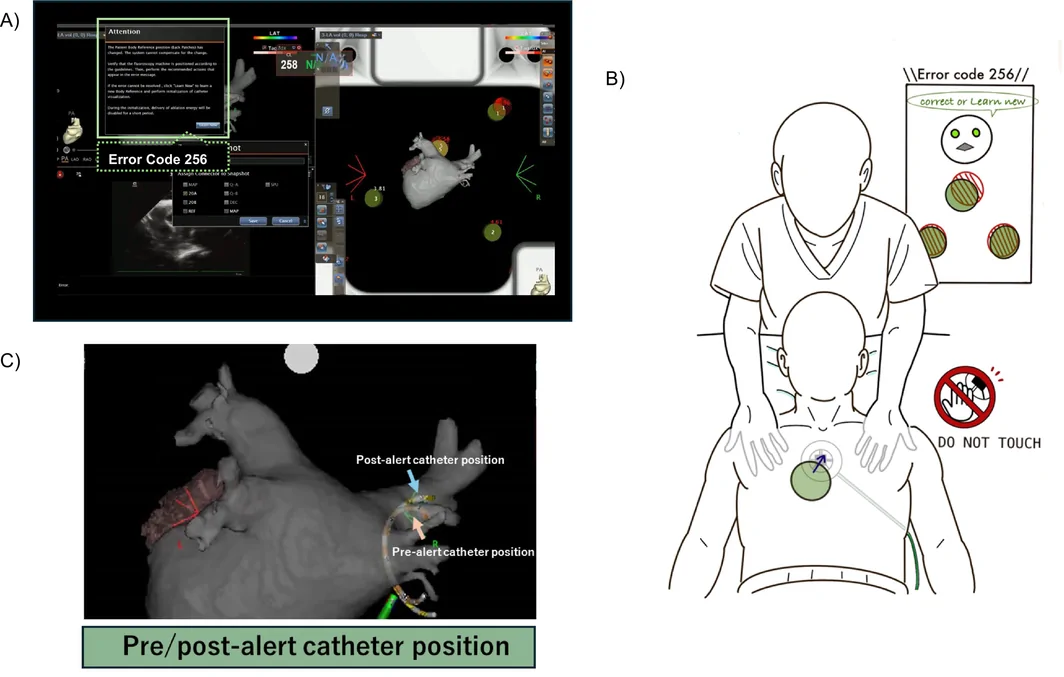
Manual Correction Technique for a Back‐Patch‐Related Positional Alert During PFA. (A) System positional alert (Error Code 256) triggered during a coughing episode. The alert was caused by displacement of the cranial‐side back patch due to cough‐induced body movement while approaching the right inferior pulmonary vein (RIPV), (B) Manual correction of back patch displacement during a coughing episode. A hand is gently inserted under the patient's back from the head side without direct contact with the patch. Light counter‐traction is applied to the skin opposite the direction of displacement. The alert resolved within seconds without using the “Learn New” function, (C) The position of the PFA catheter was marked with a shadow before the error alert appeared. After alert resolution, the catheter position appeared to closely match the pre‐alert shadow, with no obvious displacement on visual comparison. PFA = Pulsed field ablation, RIPV = Right inferior pulmonary vein.

When the positional alert occurred, we used the shortcut key Ctrl + P to visualize the reference patches in the CARTO system. The display indicated that only the cranial‐side back patch had shifted caudally relative to its baseline position. Recognizing that this displacement was caused by skin laxity and shifting rather than true detachment of the adhesive patch, we attempted to manually restore the original skin‐to‐skeleton anatomical relationship. An operator's hand was gently inserted under the patient's back from the cranial direction, explicitly avoiding direct contact with the patch to prevent further dislodgment. Light manual counter‐traction was then applied directly to the skin, pulling it in the direction opposite to the apparent displacement (cranially). The alert resolved within approximately 2 s, and the identical maneuver successfully resolved a subsequent alert episode in the same patient. After alert resolution, the real‐time catheter location was compared with the pre‐alert catheter shadow and procedural landmarks; no obvious catheter‐position mismatch was observed (Figure 1B,C; Video S1).

During the study period, positional alerts occurred in 5 of 45 patients (11.1%), and manual correction was attempted in 23 alert episodes. The alert was resolved in 22 episodes (95.7%). The single failed episode (4.3%) occurred because excessive patient perspiration reduced skin friction, preventing the operator from applying adequate manual counter‐traction. The median BMI of these five patients was 23.3 kg/m [2] (range, 20.3–30.1 kg/m [2]). Among the 22 successfully corrected episodes, the median measured positional displacement was 11.69 mm (range, 5.76–20.72 mm), and the median correction time was 1.62 s (range, 0.43–11.44 s). The displacement direction was most frequently toward the 8 o'clock direction (36.4%), followed by the 7 o'clock (31.8%), 5 o'clock (27.3%), and 4 o'clock directions (4.5%). These findings provide quantitative descriptive data on the observed alert episodes, while not establishing broad reproducibility, generalizability, or complete preservation of mapping accuracy.

When available, pre‐ and post‐correction anatomical maps were compared to assess anatomical consistency after alert resolution. No gross mismatch in left atrial or pulmonary venous anatomy was observed (Figure 2). However, this assessment was used only as supportive evidence because small localization errors cannot be excluded by visual anatomical comparison alone.

**FIGURE 2:**
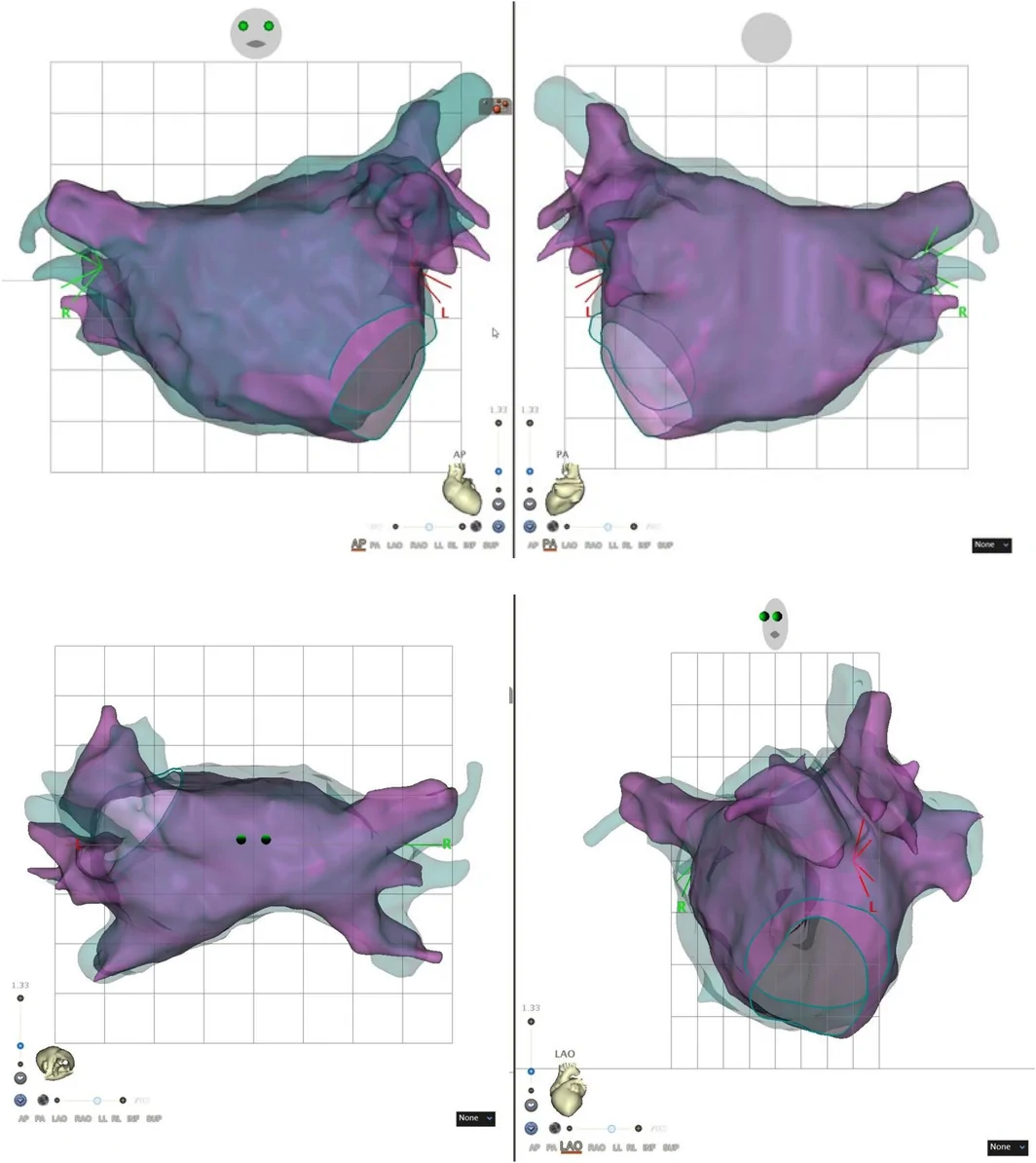
Comparison of Pre‐ and Post‐Correction Anatomical Maps Comparison of pre‐ and post‐correction anatomical maps. The green map indicates the pre‐correction anatomical map, and the pink map indicates the post‐correction anatomical map. No gross mismatch in left atrial or pulmonary venous anatomy was observed after alert resolution.

The positional alert appeared to be associated with cranial‐side back‐patch displacement during coughing or body movement. Because the detailed positional thresholds and the relative contribution of individual back‐patch sensors are not disclosed, we avoided making mechanistic conclusions regarding the exact trigger of the alert.

This report has important limitations. The maneuver was evaluated in a limited number of cases and measurements, and catheter‐location consistency was assessed by CARTO‐based measurement and visual comparison rather than by an independent quantitative localization standard. Therefore, this approach should not be interpreted as a replacement for standard reference reacquisition or as a routine troubleshooting method. Because positional alerts may be influenced by body habitus, patch adhesion, sedation depth, respiratory motion, body movement, catheter type, and mapping system settings, these findings should be interpreted as limited observational data rather than evidence of broad generalizability. Because this maneuver has not been validated by the manufacturer or by systematic safety testing, it should be attempted only cautiously in selected situations and should not delay standard correction or Learn New when safety or positional consistency is uncertain. This maneuver may not be effective in all cases, and standard correction or Learn New should be performed when the alert does not resolve promptly, when patch detachment is suspected, or when catheter‐location consistency remains uncertain.

In selected situations where a positional alert appears to result from minor cranial‐side back‐patch displacement during coughing or body movement, this simple manual maneuver may provide a practical temporary option to restore the reference relationship and preserve procedural continuity. Further cases and quantitative validation are needed before broader use can be recommended.

## Author Contributions

Conceptualization, data acquisition, and drafting: S.Y. Supervision and critical revision: T.K. Manuscript review and approval: S.Y., T.K., M.N., R.I., M.K., T.Y., S.M.

## Funding

The authors have nothing to report.

## Disclosure

The authors have nothing to report.

## Ethics Statement

The study was approved by the Institutional Review Board of Mitsubishi Kyoto Hospital. Informed consent was obtained.

## Consent

The patient provided written informed consent for the ablation procedures and agreed to the publication of her case details and images in this report.

## Conflicts of Interest

The authors declare no conflicts of interest.

## Supporting information


**Video S1:** A coughing episode during PFA caused cranial‐side back patch displacement and triggered a positional alert (Error Code 256). The alert was resolved within seconds using the described manual technique.

## Data Availability

All data generated or analyzed during this study are included in this published article.
